# Naval Discipline

**Published:** 1869-10

**Authors:** 


### Naval Dicipline.

S/

The Navy department has lately promulgated in general orders the result of a general court-martial, convened at the Washington Navy Yard, for the trial of Passed Assistant Surgeon, Charles L. Greene. The finding of the said court is not only of great interest to the medical staff of the service, but to the profession at large. It carries with it a selfish and partial exemplification of a principle which those who may have a desire to enter the navy as surgeons should gravely contemplate. The following were the charges:—

"*First*—Treating with contempt his superior officer while in the execution of his office, in dissenting, while on board the United States steamer, Nipsic at Aspinwall, from an order to take a seaman off the sick list.

"*Second*—Disobeying a lawful order of his superior officer in thus refusing obedience.

*Third*—Conduct unbecoming an officer, the specifications being that he was intoxicated and behaved in a manner unbecoming an officer in the town of Aspinwall."

After an *impartial* examination into the facts of the case, by an *impartial* jury, the surgeon was found giulty of the first two. The doctor had coscientiously refused to take a seaman off the sick list, because, in his opinion as a medical man, the said seaman was physically incapable of performing his duties. He dared do this despite the order of a line officer who was his superior in rank, and who, the charge specifies, was at the time "in the execution of his office." This order is also particularized as a lawful one, hence the contempt of authority which entitled the unfortunate surgeon to a trial.

The result of the trial clearly implies a divine right on the part of a line officer not only to be superior in rank to the surgeon of his ship, but also his superior in medical knowledge. The profession have been prepared to hear of anything in the shape of indignity being heaped upon the friendless ship-surgeon, but we imagine that this new interpretation of dicipline will be rather startling. A medical gentleman has not an enviable lot in any community; it is generally summed up in hard work and poor pay, but he has reason to believe that his knowledge of his profession always associates with it a respect for his medical opinions. By the common consent of civilized communities his judgement in his special department is at a premium, and none of the laity, so-called, deem it safe from their standpoint of knowledge to question its integrity. Everywhere, save in the naval service of the United States, a medical man is supposed to know the most of medical science, and to be the best judge as to physical ability or disability.

The names of the gentlemen composing the court material do not appear, but it is quite evident, from the decision arrived at, that the majority at least were made up from members of the line. Indeed this is the only way of explaining the verdict. Nothing but that bitterness of hate which the line bears towards the staff could have prompted a decission so contrary to the ordinary dictates of common sense. But after all, what is a naval surgeon? His rights to be even a gentleman are only conceeded to him out of gracious courtesy, and by and by he will not dare to hold a medical opinion, except by express command of his superior officer.

After having been declared guilty of the first two charges and specifications, the court sentenced the surgeon to be suspended from rank, on furlough pay, for the term of two years, and to be publicly reprimanded by the Honorable Secretary of the Navy, the order reprimanding him to be read to the officers and men of each naval station and vessel in commission.

On examination of the evidence, and after considerable deliberation, Secretary Robeson approved the finding of the court. With an attempt to show a charity towards the poor offender, the Honorable Secretary let him off with a simple reprimand in general orders, but the excuse for doing even this is perhaps more remarkable than the actual sentence itself. "Thus learnedly and eloquently spoke the judge:"—

"The sentence of the court is not unsuited to the offence of which the accused was found guilty. Disobedience of orders is under any circumstance a serious offence, and when committed deliberately by an intelligent officer, under a claim of right, must tend greatly to the subversion of all dicipline. I am inclined to think, however, from the evidence, that the disobedience complained of in this case was the result of a mistake of judgement in regard to professional rights and duties, rather than of a deliberate intention of wrong. Mistakes of this kind rarely require a severe, and never a disgraceful punishment, and the previous good character and conduct of Mr. Green entitled him to the benefit of whatever doubt there may be on this subject, and to the consideration of the reviewing authority. The sentence of suspension from rank on furlough pay for the term of two years is, therefore, remitted, and this order is published as the reprimand provided for in the sentence and it will be read accordingly."

A more emphatic and explicit endorsement of the spirit of any sentence, just or unjust, could not have been imagined. Coming as it does from the head of the Department, there is virtually no appeal. Obedience of naval orders implies to the naval surgeon who is graciously named an "intelligent officer," the right of possessing no judgement whatever in regard to professional rights or professional opinion when the requisite order comes to him from his superior. If a man is hereafter ordered to be taken from the sick list, no matter what his state may be, he must become well at once, and fit for duty, otherwise there will be "a subversion of all dicipline;" and if the surgeon, as the lawful custodian of the health of the ship, has reason to believe that the patient is physically incapable, and for the sake of the sick one refuses to say that he is well enough to attend to his duties, the offence against dicipline is the more flagrant because the surgeon is called an "intelligent officer."

Our readers have had, we trust, a tolerable idea of the position of a naval surgeon on shipboard. He has relatively been a nobody; but now he is a positive nonentity. He has been merely a creature of the coldest sort of courtesy; but now he has no right as a medical man to have even a medical opinion independent of his commander.

There have been forcible arguments to prove the necessity for absolute staff rank in the navy; but the definition of professional rights by this naval court-martial makes a claim stronger than all the rest. The framers of the Naval Staff Rank Bill, with all their knowledge of the rights which might be claimed by the line, could not have dreamed of the necessity of defining purely professional prerogatives. Surgeon Green, by the noble stand he has taken for his profession, has demonstrated to them the necessity for such an additional provision.

We have taken occasion to remark on this matter of naval discipline for the purpose not only of heartily endorsing the conduct of Passed Assistant Surgeon Green, but of keeping the profession at large informed as to the indignities sufferred by their brethern in that branch of the service. It would seem that the good offices of our profession are not very much needed in the navy But we must not mourn over this. In every other sphere the properly educated and "intelligent" medical man can be respected, nay, honored; can command an influence in proportion to his professional acquirements, and, if hard pinched, can, by dint of industry and frugality, gain a livelihood. It may, after all, in time be proved that the profession can do better without the help of the navy than with it. It certainly seems so at present. In the army, however, the treatment of the medical officer is so different that one would hardly believe the two branches of the service to belong to the same country. It is this later circumstance, we imagine, which can explain why the army has so few vacancies in the medical corps, and the navy so many. This disparity must inevitably continue to be greater as the antagonism of the line towards the staff becomes more generally understod and appreciated.—*New York Med. Reporter.*

